# Cocrystals Enhance Biopharmaceutical and Antimicrobial Properties of Norfloxacin

**DOI:** 10.3390/pharmaceutics15092211

**Published:** 2023-08-26

**Authors:** Samantha Nascimento Gomes, Isabela Fanelli Barreto Biscaia, Diana Schon Lopes, Mariana Mengarda, Fábio Seigi Murakami, Paulo Renato Oliveira, Larissa Sakis Bernardi

**Affiliations:** 1 Graduate Program in Pharmaceutical Sciences, Department of Pharmacy, Universidade Estadual do Centro-Oeste (UNICENTRO), Guarapuava 85040-080, Brazil; samanthangomes@gmail.com (S.N.G.); isabelafbarreto@hotmail.com (I.F.B.B.); dianaschonlopes@gmail.com (D.S.L.); larissa.sb@gmail.com (L.S.B.); 2 Graduate Program in Pharmaceutical Sciences, Department of Pharmacy, Universidade Federal do Paraná (UFPR), Curitiba 80210-170, Brazilfsmurakami@ufpr.br (F.S.M.)

**Keywords:** cocrystal, norfloxacin, colorimetric microdilution assay

## Abstract

A solvate cocrystal of the antimicrobial norfloxacin (NFX) was formed by using isonicotinamide (INA) as a coformer with the solvent evaporation technique. The cocrystal formation was confirmed by performing solid-state characterization techniques. We evaluated the dissolution under supersaturated conditions and also the solubility at the vertex of triphasic domain of cocrystal and NFX in both water and Fasted-State Simulated Intestinal Fluid (FaSSIF). The antimicrobial activity was evaluated using the microdilution technique. The cocrystal showed 1.8 times higher dissolution than NFX in water at 60 min and 1.3 times higher in FaSSIF at 180 min in the kinetic study. The cocrystal also had an increase in solubility of 8.38 times in water and 6.41 times in FaSSIF. The biopharmaceutical properties of NFX with cocrystallization improved antimicrobial action, as shown in the results of minimum inhibitory concentration (MIC) and inhibitory concentrations of 50% (IC50%) and 90% (IC90%). This paper presents, for the first time, a more in-depth analysis of the cocrystal of NFX–INA concerning its dissolution, solubility, and antimicrobial activity. In all these criteria, the cocrystal obtained better results compared to the pure drug.

## 1. Introduction

Molecules with limited aqueous solubility are often the target of diverse research because they exhibit slow dissolution in biological fluids, insufficient systemic exposure, and reduced efficacy in patients [1]. Thus, one of the main challenges in the pharmaceutical industry is to improve the solubility of drugs [2,3]. The development of new pharmaceutical formulations aims to improve the handling of ingredients during processing, improve stability, and increase drug dissolution [4]. Chemical changes are very common in solid-state molecules as they allow changes in the physical properties of a drug without changing its mechanism of action. In some multicomponent systems, such as salts and cocrystals, the interactions formed are of a non-covalent nature and, therefore, of the supramolecular type [5].

In recent years, insight into the pharmaceutical engineering of cocrystals has gained high visibility [6]. There has been increased interest in pharmaceutical cocrystals, which is a robust method for increasing the solubility and bioavailability of poorly soluble drugs [2]. Cocrystals are structurally homogeneous crystalline materials formed by at least two neutral compounds that are found in defined stoichiometric quantities and are solid at ambient temperature [7]. A pharmaceutical cocrystal is composed of an active pharmaceutical ingredient (API) and a coformer, which can be another drug or a non-toxic molecule [2]. Research proves the achievement of higher solubility and stability of drugs from cocrystals, which is why it is possible to state that this is a reliable method to change the physical properties of drugs without modifying their pharmacology [8].

**Figure 1:**
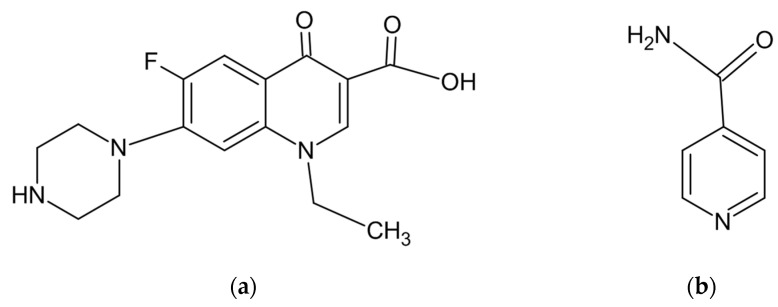
Chemical structures of norfloxacin (**a**) and isonicotinamide (**b**).

NFX (Figure 1) is a synthetic second-generation fluoroquinolone molecule intended for use as a broad-spectrum antibacterial drug for urinary tract infections, including cystitis and prostatitis [9,10]. According to the biopharmaceutical classification system, NFX fits as a schedule IV drug. Its low solubility and low permeability characterize a serious bioavailability problem. Only 30–40% of orally administered NFX is absorbed after administration of 400 mg, yielding a maximum plasma concentration (Cmax) of approximately 1.5 μg mL^−1^ in humans [11,12,13].

There are reports in the literature of the formation of some NFX cocrystals, such as a cocrystal solvated with isonicotinamide/chloroform [14], a salt cocrystal with saccharin [15], a dual-drug cocrystal with ciprofloxacin [16] a cocrystal with riboflavin [17], another with resorcinol [18], an NFX cocrystal with two known isomers of nicotinic acid (picolinic acid and isonicotinic acid) [19], and a cocrystal with nicotinamide, cinnamic acid, and sorbic acid [20]. All these NFX crystals cited are listed in more detail in Table 1. The stability mentioned in Table 1 was based on the comparison of the melting point of the NFX and the cocrystal. If the melting point of the cocrystal is higher than the pure drug, it is considered more stable. This way, a lower melting point for the cocrystal indicates a less stable compound.

Although microbial resistance involves a natural expression of bacterial evolution and genetics, widespread and abusive use of antimicrobials can accelerate this process [21,22]. With its steady increase, microbial resistance has become a public health problem and a global threat [23,24,25]. Due to the difficulty in obtaining new compounds with antimicrobial activity [26], the use of chemical alterations becomes essential; this allows changes in the physical properties of a drug without changing its mechanism of action [5]. One way to change the properties of API is the addition of a second component to the formulation, generating a salt or cocrystal [27]. Restricting access to antibiotics and expanding patient knowledge about antimicrobial resistance are some measures that seek to improve rational antibiotic use and reduce adverse drug reaction rates [28].

The most important methods to evaluate the activity of antimicrobials against microorganisms in vitro are dilution methods, which include macrodilution and microdilution. The substances are added to a liquid culture medium, where the microorganism to be tested is inoculated. After the incubation period, the growth can be determined directly by visual reading or spectrophotometry [29].

Microdilution techniques are considered quantitative because they can determine the MIC, which is the lowest concentration of antimicrobial agent capable of visibly inhibiting the growth of microorganisms [30]. There are several advantages to using microdilution, such as sensitivity, reproducibility, the convenience of having commercial plates prepared with antibiotics, space and reagent savings, and the possibility of using automated reading systems to facilitate reporting [31,32]. Such advantages have led to the wide use of this technique in determining the MIC of compounds with potential antimicrobial activity [31,33,34,35,36,37,38].

We sought to improve the biopharmaceutical properties of this drug through the formation of NFX cocrystals. The objective was to improve factors like dissolution, solubility, and antimicrobial properties, which were evaluated by colorimetric microdilution assay.

## 2. Materials and Methods

### 2.1. Materials

NFX (purity > 99.8%) was obtained from Via Farma (São Paulo, Brazil). Isonicotinamide (purity > 99%) and acetonitrile (HPLC, purity > 99.5%) were obtained from Sigma Aldrich (Stockholm, Sweden). Chloroform (P.A. purity > 99.8%) was obtained from Labsynth (São Paulo, Brasil).

### 2.2. Obtaining Norfloxacin–Isonicotinamide Cocrystal

Norfloxacin–isonicotinamide–chloroform solvatade cocrystals were obtained via the solvent evaporation technique, using a 1:1:1 molar ratio of dried drug, coformer, and solvent (31.9 mg of NFX and 12.2 mg of INA diluted in 8 mL of chloroform) in a 25 mL Erlenmeyer flask, based on a previous method already described in the literature [14]. The chloroform was left to evaporate in a water bath (SP Labor, São Paulo, Brazil) with a controlled temperature of 30 °C to prevent the external temperature from interfering with the solvent evaporation time and, consequently, the cocrystal formation [39].

### 2.3. Solid State Characterization Techniques

#### 2.3.1. X-ray Powder Diffraction (XRPD)

XRPD patterns were collected on a D2 Phaser X-ray diffractometer (Bruker Corporation, Billerica, MA, USA). The diffraction patterns were obtained at a voltage of 30 kV and current of 10 mA, CuKα radiation, λ = 1.5418 Å. X-ray scanning was performed at 2θ open angle between 5° and 40°, with a 1 s pass time and 0.05° increment. The samples were kept at 5 rpm during the analysis.

#### 2.3.2. Fourier Transform Infrared Spectroscopy (FTIR)

The FTIR spectrum was obtained on Prestige FTIR equipment (Shimadzu, Kyoto, Japan) within a scan range of 4000–600 cm^−1^, averaged over 32 scans, and with a spectral resolution of 4 cm^−1^ using the attenuated total reflection (ATR) technique. A background (blank) spectrum was performed.

#### 2.3.3. Differential Scanning Calorimetry (DSC)

DSC curves were performed on a DSC-60 cell (Shimadzu, Kyoto, Japan). Partially closed aluminum crucibles, subjected to a dynamic atmosphere of N_2_ (100 mL min^−1^), were used, with a heating rate of 10 °C min^−1^, in the temperature range of 30 to 300 °C. The equipment was previously calibrated with indium (melting point = 156.6 °C; ∆H melting = 28.54 J/g) and zinc (melting point = 419.6 °C). The observed melting points in the DSC analysis were confirmed in a PMF II melting point meter (Digilab, São Paulo, Brazil).

#### 2.3.4. Thermogravimetry (TG)

A TGA-50 thermobalance (Shimadzu, Kyoto, Japan) was used. Alumina crucibles containing the sample were subjected to a heating rate of 10 °C min^−1^ over a temperature range of 30 to 300 °C in a dynamic N_2_ atmosphere (100 mL min^−1^).

#### 2.3.5. Scanning Electron Microscopy (SEM)

Photomicrographs were taken on a Vega 3 model scanning electron microscope (Tescan, Bruno, Czech Republic) with an Everhart–Thornley-type secondary electron detector (SE), with a positive potential front grid and a 5 kV voltage filament. NFX and cocrystal photomicrographs were taken at 500× magnification, and INA photomicrographs were taken at 460× magnification.

### 2.4. Analysis of Cocrystal Dissolution under Supersaturation Conditions

This kinetic method, performed in water and FaSSIF, determines cocrystal dissolution using dynamic process time scales and drug concentration fluctuations during dissolution [40,41,42]. All analyses were performed in triplicate and the statistical analysis was performed using Student’s *t* Test, which evaluated the statistical significance of the AUCs of the dissolution profiles using STATISTICA^®^ software version 8.0.

In the study, excess drug and cocrystal were added to 250 mL capacity flasks containing 100 mL of medium and kept under orbital agitation in a Shaker Incubator nl-343-01 (New Lab, São Paulo, Brazil) at 110 rpm. The temperature was maintained at 25.0 ± 0.2°C for analysis in water and 37.0 ± 0.2°C for analysis in FaSSIF. Both studies had a duration of 4 h, with aliquots collected at time intervals of 1; 2.5; 5; 7.5; 15; 30; 40; 50; 60; 90; 120; 150; 180; 210; and 240 min. These aliquots were filtered through a 0.45 μm Nylon syringe filter (Sterlitech, Auburn, WA, USA), diluted, and analyzed by high-performance liquid chromatography (HPLC) (Shimadzu, Kyoto, Japan).

The bio-relevant medium simulating the intestinal fluid in the fasting state presents the following composition: sodium taurocholate (3 mM); soy lecithin (0.75 mM); sodium chloride (105.9 mM); sodium hydroxide (8.7 mM); and monobasic sodium phosphate (28.4 mM), with osmolarity (mOsm kg^−1^) 270 ± 10 and pH 6.5 [43].

For the analysis in HPLC of NFX, the parameters followed the descriptions by Oliveira et al. [44]. The chromtographic column used was a Luna^®^ C_18_ 150 × 4.6 mm column, with the mobile phase composed of acetonitrile: phosphoric acid 0.04 M pH 3.0 (16:83 v/v) eluted in isocratic mode. The flow rate was 1.0 mL min^−1^ with an injection volume of 20 µL. The oven temperature was 40 °C, and the detection wavelength was at 272 nm with a run time of 6 min [44].

The dissolution profiles were obtained, and the area under the curve (AUC) was calculated, which was used as a general measure for each profile. Student’s *t* Test was used to test the statistical significance of the AUCs of the dissolution profiles. Differences were considered significant at *p* < 0.05, with a confidence level of 95%. The results were analyzed using STATISTICA^®^ software version 8.0.

### 2.5. Solubility of the Cocrystal at the Vertex of Triphasic Domain and Eutectic Constant (Keu)

Solubility studies at the vertex of triphasic domain were performed in ultrapure water and FaSSIF, following the methodology described by Good and Hornedo and Kuminek et al. [41,45].

#### 2.5.1. Solubility of the Cocrystal at the Vertex of Triphasic Domain in Water

For this study, two Erlenmeyer flasks were used, identified as “Water Sample 1 (48 h)” and “Water Sample 2 (72 h)”, each containing 10 mL of ultrapure water. In each of these two flasks, 500 mg of cocrystal and 50 mg of NFX were added. They both were left under stirring at 110 rpm at 25.0 ± 0.2 °C in a Shaker Incubator with orbital agitation nl-343-01 (New Lab, São Paulo, Brazil). After 48 h, “Water Sample 1 (48 h)” was removed from the shaker, and its content was filtered on a quantitative filter paper with a pore size of 45 µm. The liquid phase had its pH evaluated and was subjected to HPLC (Shimadzu, Kyoto, Japan) to measure the concentrations of both drug and coformer at equilibrium, while the solid phase was maintained in a desiccator containing silica gel to remove moisture and continue the XRPD analysis. Then, 72 h after the start of the experiment, the second Erlenmeyer, containing “Water Sample 2 (72 h)”, was removed from the Shaker and subjected to the same process as the previous sample. The solid phases that were collected from Samples 1 and 2 remained in the desiccator for three days so that they remained completely dry and could be analyzed in XRPD in a D2 Phaser X-ray diffractometer (Bruker Corporation, Billerica, MA, USA) to verify the presence of NFX and cocrystal, to prove that the vertex of triphasic domain was reached.

#### 2.5.2. Solubility of the Cocrystal at the Vertex of Triphasic Domain in FaSSIF

For this study, two Erlenmeyer flasks were used, identified as “FaSSIF Sample 1 (48 h)” and “FaSSIF Sample 2 (72 h)”, each containing 10 mL of FaSSIF. In each of these two flasks, 1 g of cocrystal and 100 mg of NFX were added. They both were left under stirring at 110 rpm at 37.0 ± 0.2 °C in a Shaker Incubator with orbital agitation nl-343-01 (New Lab, São Paulo, Brazil). After 48 h, the “FaSSIF Sample 1 (48 h)” was removed from the shaker, and its content was filtered on a quantitative filter paper with a pore size of 45 µm. The liquid phase had its pH evaluated and was subjected to HPLC (Shimadzu, Kyoto, Japan) to measure the concentrations of both drug and coformer at equilibrium, while the solid phase was maintained in a desiccator containing silica gel to remove moisture and continue the XRPD analyses. After 72 h from the start of the experiment, the second Erlenmeyer, containing “FaSSIF Sample 2 (72 h)”, was removed from the Shaker and subjected to the same process as the previous sample. The solid phases that were collected from Samples 1 and 2 remained in the desiccator for three days so that they remained completely dry and could be remanufactured in XRPD in a D2 Phaser X-ray diffractometer (Bruker Corporation, Billerica, MA, USA) to verify the presence of NFX and cocrystal, to prove that the vertex of triphasic domain was reached.

#### 2.5.3. Calculation of Cocrystal Solubility at the Vertex of Triphasic Domain and Eutectic Constant (Keu)

The solubility of the cocrystal at the vertex of triphasic domain was calculated by the vertex solution concentrations of the drug and the coformer for a 1:1 as per Equation (1), while the solubility advantage (SA) was calculated by dividing the solubility of the cocrystal by the solubility of the NFX, as per Equation (2) [45].

(1) $$S_{cocrystal}^{1:1}=\surd {\left[drug\right]}_{eu,\;T}{\left[coformer\right]}_{eu,T}$$

(2) $$SA=\frac{S_{cocrystal}}{S_{drug}}$$

It is also possible to obtain a valuable indicator of the solubility and the stability of the cocrystal by determining the vertex of a triphasic domain: the eutectic constant (*K_eu_*). *K_eu_* is represented by the ratio of the activities of the coformer and the drug (a) at the vertex of a triphasic domain, which can be approximated to the ratio of the concentrations, as shown in Equation (3) [45]:

(3) $$K_{eu}={\left(\frac{S_{cocrystal}}{S_{drug}}\right)}^{2}$$

When dealing with a cocrystal of stoichiometric ratio 1:1, a *K_eu_* > 1 indicates that the cocrystal is thermodynamically unstable, which represents a higher solubility, while a *K_eu_* < 1 is indicative of higher thermodynamic stability and, consequently, a lower solubility of the cocrystal in comparison to the drug [45,46].

### 2.6. Colorimetric Microdilution Assay

#### 2.6.1. Determination of the Minimum Inhibitory Concentration (MIC) of Antimicrobial Compounds

The MIC was determined using the technique described by Veiga and colleagues [29]. NFX, cocrystal (COC), and the physical mixture of NFX with INA (PM) were evaluated, and standard strains of *Escherichia coli* (ATCC 8739), *Pseudomonas aeruginosa* (ATCC 9027), and *Staphylococcus aureus* (ATCC 6538) were used. The microorganisms were determined as described by the Brazilian Pharmacopeia (6th edition) for antimicrobial efficacy testing.

The freeze-dried microorganisms were revitalized in tryptone soy broth (TSB). After incubation at 35 °C/24 h, depletion culture was performed on tryptone soy agar (TSA) at 35 °C/24 h. After incubation, the strains were passed into tubes containing 0.9% NaCl saline solution, where turbidity was adjusted by visual comparison with a tube corresponding to 0.5 on the McFarland Scale (1.5 × 10^8^ CFU/mL) using a V.2AW suspension turbidity detector densitometer (Biosan, Riga, Latvia). NFX, COC, and PM samples were prepared at a concentration of 10 µg mL^−1^ using sterile purified water. For the positive control, the antimicrobial chloramphenicol was used at a concentration of 100 µg mL^−1^.

The assays were performed in 96-well microplates with a U-shaped bottom. Only one microorganism was tested on each plate to avoid cross-contamination. Previously, 100 μL of Mueller Hinton broth was added to all the wells that were used. Then, in triplicate, 100 μL of the samples to be tested were added to the holes in row A, and serial dilutions were performed. As blank, all the components of the other wells (100 µL of Mueller Hinton broth and 100 µL of the samples to be tested) were used, except the microorganism. For the negative control blank, 100 µL of Mueller Hinton broth and 100 µL of sterile water were used; however, the same volume that would be added of microbial suspension was added of saline solution (10 µL). As a negative control, 100 μL of Mueller Hinton broth and 100 μL of sterile water were used. As for the positive control, a solution of Chloramphenicol 100 μg mL^−1^ in a volume of 100 μL was used to inhibit the growth of bacteria. Then, 10 μL of the microorganism suspension was added to each well, except the wells corresponding to the blanks.

The plates containing the bacteria were incubated at 35°C ± 0.5°C for 22 h. After incubation, 20 μL of 0.125% 2,3,5-triphenyltetrazolium chloride (TTC) solution was added to all holes, and the plates were again incubated at 35°C ± 0.5 °C for 2 h. The MIC was determined by visual reading of the microplates, considering the last concentration, in triplicate, in which there was no development of red coloration for each of the microorganisms.

#### 2.6.2. Determination of the Inhibitory Concentration of 50% (IC50%) and 90% (IC90%) of the Microorganisms

As described by Veiga and co-workers [29], two hours after the addition of the 0.125% TTC solution, absorbances were read in a microplate photometer at 540 nm (Multiskan FC, Thermo Fisher Scientific, Waltham, MA, USA). The absorbance values were applied to the linearity equations already standardized for each of the microorganisms according to the study by Veiga and collaborators, where *y =* 1 × 10 − 8*x* + 0.0097 for *S. aureus*; *y* = 3 × 10 − 9*x +* 0.0223 for *E. coli*; and *y =* 1 × 10 *−* 8*x* + 0.024 for *P. aeruginosa*, where y is equal to the absorbance value at 540 nm and x is equal to CFU mL^−1^. Then, the CFU mL^−1^ values were treated with a logarithmic function to construct a new growth curve, correlating the log of CFU mL^−1^ with the antimicrobial concentrations tested.

For determining the IC50% and IC90%, the CFU mL^−1^ values from the negative control wells were multiplied by 0.5 and 0.1 to obtain the number of microorganisms corresponding to 50% and 10% of the total growth. The values were then transformed by logarithmic function and applied as the value of *x* in the straight-line equations constructed from the correlation between antimicrobial concentration and the log CFU mL^−1^ of the microorganisms.

## 3. Results

### 3.1. Solid State Characterization Techniques

**Figure 2:**
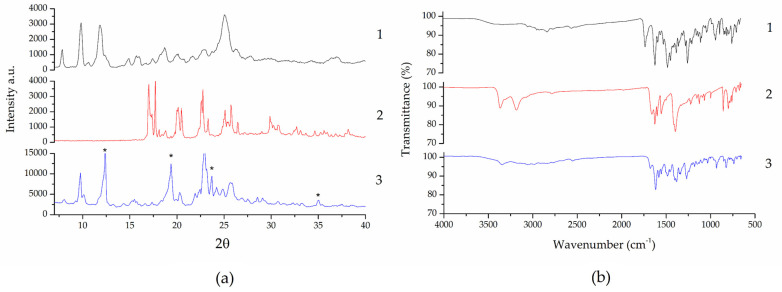
XRPD (**a**) and FTIR (**b**) spectra of NFX (1), INA (2), and cocrystal (3). * Diffraction peaks at 12.24°, 19.36°; 23.72°, and 35.04° are characteristic of the cocrystal.

Figure 2 shows results for X-ray powder diffraction (XRPD) (a) and Fourier-transform infrared spectroscopy (FTIR) (b). In Figure 2a, the XRPD spectra show that the cocrystal sample presented diffraction peaks that could not be observed in the NFX nor in the INA coformer, which indicates the formation of a new crystalline structure. Such diffraction peaks were observed at 12.24 °, 19.36°, 23.72°, and 35.04°.

**Figure 3:**
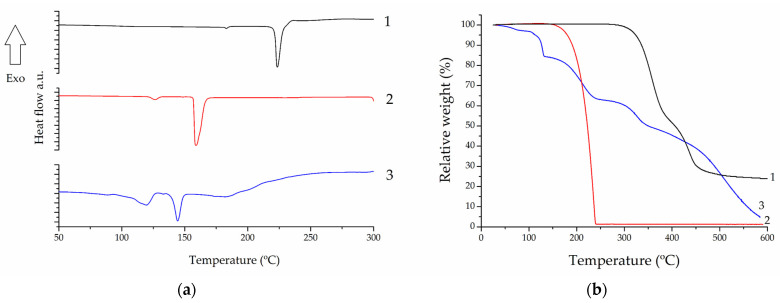
DSC (**a**) and TG (**b**) curves of NFX (1), INA (2), and cocrystal (3).

In Figure 2b, the cocrystal spectrum showed that the band present at 1727 cm^−1^ in NFX disappears, possibly due to the interaction between the carbonyl oxygen in the carboxylic acid in NFX with the hydrogen in the amide in the coformer INA [14] or still due to the bands of the INA coformer in this region (1651 cm^−1^). At 1582 cm^−1^, we observed a band in NFX that indicates that it is in its zwitterionic form due to the presence of adsorbed water molecules (a weight loss due to adsorbed water can be observed in the TG results of NFX, Figure 3b). The same band in the cocrystal shows that NFX remains in its zwitterionic form, which may be due to adsorbed water or the presence of the solvent used to obtain it (CHCl_3_), as suggested by Basavoju et al. [14].

We can also observe a peak in the cocrystal at 3344 cm^−1^. This is due to both the interaction between the oxygen in carbonyl from the carboxylic acid in NFX with the hydrogen in the amine from the INA coformer and the interaction of the oxygen in the carbonyl in INA with the hydrogen in the amine in another INA molecule. The cocrystal forms an amide–amide homodimer synthon, which confirms that there was indeed cocrystal formation [14].

Analyzing the results obtained for NFX in the DSC (Figure 3a) and TG (Figure 3b) curves, a small endothermic event was observed at T_onset_ = 181.2 °C (T_peak_ = 182.9 °C), followed by an endothermic event at T_onset_ = 221.5 °C (T_peak_ = 223.5 °C). The first event corresponds to the solid–solid transition from polymorph B to NFX in polymorph A, a temperature at which no mass loss is observed in TG. The second event corresponds to the melting of NFX [47], from which the occurrence of three thermal mass loss events related to drug degradation are visible in TG (the first event occurs at 295 °C, the second at 389 °C, and the third at 505 °C) [48].

The DSC/TG results obtained for the coformer showed two endothermic events at T_onset_ = 122.0 °C (T_peak_ = 126.4 °C) and T_onset_ = 156.9 °C (T_peak_ = 158.8 °C): the first refers to the solid–solid transition from the INA2 or INA3 polymorphs to the INA1 polymorph, where no mass loss is observed in TG; the second corresponds to the sublimation of the coformer, from which a mass loss of degradation in a single step is observed, at around 156 °C [49].

Regarding the cocrystal, we can observe in the DSC a small endothermic event at T_onset_ = 110.0 °C (T_peak_ = 119.5 °C), which is followed in the TG curve by a mass loss of approximately 20%, consistent with the desolvation of the cocrystal by evaporation of the chloroform molecule present in its structure. The second and major endothermic event at T_onset_ = 140.5 °C (T_peak_ = 144.2 °C) corresponds to the melting point of the cocrystal, being a congruent melting point peak. A broad endothermic event is also observed at T_onset_ = 179.5 °C (T_peak_ = 182.2 °C), which corresponds to the degradation of the cocrystal after melting as also observed in the mass loss of the TG curve at 160 °C, 280 °C, 386 °C, and 454 °C.

The melting points of the three analyzed samples were confirmed using a PFM II melting point meter (Digilab, São Paulo, Brazil). The observed results were MP_NFX_ = 223 °C, MP_INA_ = 155 °C, and MP_COC_ = 143 °C.

By means of SEM analysis, the differences between the NFX, INA, and COC particles were visually verified. This methodology cannot be analyzed separately for cocrystal identification, but rather must be analyzed in a complementary manner to the other solid-state characterization analyses. Photomicrographs are presented as Supplementary Material.

### 3.2. Analysis of Cocrystal Dissolution under Supersaturation Conditions

**Figure 4:**
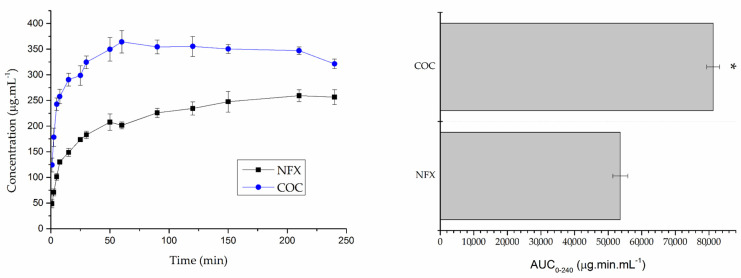
Dissolution and AUC of norfloxacin (NFX) and norfloxacin–isonicotinamide cocrystal (COC) in water. * Indicates statistically different result when compared to NFX (*p* < 0.05).

In the kinetic study in water (Figure 4), the graphs of NFX and cocrystal showed that, at 60 min, there was a maximum difference between the two dissolutions. The NFX from the cocrystal showed a dissolution of 364 μg mL^−1^, and the pure NFX showed a dissolution of 201 μg mL^−1^, indicating that, in water, the cocrystal dissolved 1.8 times more than NFX.

**Figure 5:**
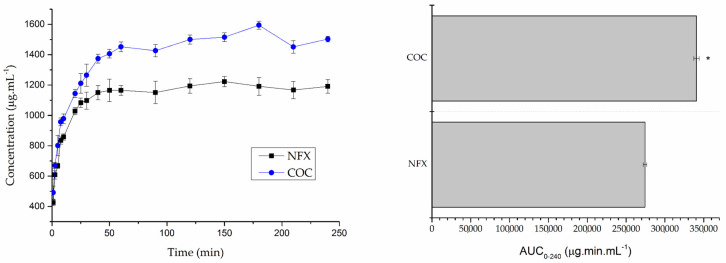
Dissolution and AUC of norfloxacin (NFX) and norfloxacin–isonicotinamide cocrystal (COC) in FaSSIF. * Indicates statistically different result when compared to NFX (*p* < 0.05).

In the FaSSIF analysis (Figure 5), at 180 min, the maximum difference between the two dissolutions can be seen. At this time, there was a dissolution of 1.594 μg mL^−1^ of NFX from the cocrystal and 1.191 μg mL^−1^ of NFX, which corresponds to a 1.3 times higher dissolution of cocrystal when compared to NFX.

### 3.3. Solubility of the Cocrystal at the Vertex of Triphasic Domain and Eutectic Constant (K_eu_)

When the vertex of triphasic domain is reached, the cocrystal and the drug should be present in both the solid phase analyzed by XRPD and the liquid phase analyzed by HPLC [44]. The XRPD results obtained in water present diffraction peaks at 9.28° and 19.36°, which are characteristics of the cocrystal pattern, and diffraction peaks at 21.64° and 27.86°, which are characteristics of the NFX pattern. The XRPD results obtained in FaSSIF present diffraction peaks at 19.36° and 21.92°, which are characteristics of the cocrystal pattern, and a diffraction peak at 10.59°, which is a characteristic of the NFX pattern. All diffraction peaks were present in both the 48 h sample and the 72 h sample in both media analyzed, indicating that both cocrystal and NFX are present in the precipitates and that the vertex of triphasic domain has been reached [41]. Solid phase XRPD plots of the vertex of triphasic domain evaluation are available as Supplementary Material.

The measurement of the concentrations of both the drug and the coformer in the equilibrium [NFX]_vtd_ and [INA]_vtd_ present in the filtered solution was performed in HPLC. The variation between the 48 and 72 h concentrations was less than 5%, indicating equilibrium of the suspension. The cocrystal solubility and solubility advantage were calculated according to Equations (1) and (2), respectively, and the pH at the vertex of the triphasic domain was also confirmed. Table 2 shows the results [41,42].

The *K_eu_* calculation was used, as described in Equation (3), to evaluate the solubility and stability of the cocrystal relative to the pure drug. The results showed that, in water, *K_eu_* obtained a value of 70.2, while, in FaSSIF, the value was 41.1.

### 3.4. Colorimetric Microdilution Assay

#### 3.4.1. Determination of the Minimum Inhibitory Concentration (MIC) of Antimicrobial Compounds

The MICs were determined by visual readings considering the last concentration without development of pink coloration, which resulted from the reduction in TTC by microbial metabolism. Table 3 shows the results of the MICs, and the images of the corresponding plates of each microorganism tested are available as Supplementary Material.

#### 3.4.2. Determination of the Inhibitory Concentration of 50% (IC50%) and 90% (IC90%) of the Microorganisms

Through the treatment with logarithmic function of the number of microorganisms corresponding to 50% and 10% of the total growth, the equations of the straight line were used to determine CI50% and CI90% for NFX, COC, and PM, which are presented in Table 4. The growth graphs with the corresponding line equations are available as Supplementary Material.

## 4. Discussion

By using solid-state characterization techniques (XRPD, FTIR, DSC, TG, and SEM), we confirmed that the pharmaceutical solvatade cocrystal of NFX–INA–CHCl_3_ had been formed. The result of the DSC curve showed a characteristic melting point peak of the cocrystal at approximately 144 °C, which did not correspond to the melting point between 180–185°C found by the authors who developed the cocrystal [14]. Therefore, it was necessary to use the melting point meter PFM II (Digilab, São Paulo, Brazil) to confirm the melting point of the cocrystal, which confirmed the melting of the NFX–INA cocrystal at 144 °C, corroborating the result observed in the DSC curve.

Dissolution is defined as the transfer of molecules or ions from a solid state into solution, and it can be assessed using drug concentration fluctuations on dynamic process time scales [41,42]. The analysis of cocrystal dissolution under supersaturated conditions proved advantageous compared to NFX in both water and FaSSIF. It is worth noting that, when adding a cocrystal to a solution and measuring drug concentration as a function of time, important properties of the cocrystal may be overlooked and lead to flaws and inaccuracies in the evaluation of its performance. For this reason, kinetic studies should not be used as an assessor of cocrystal solubility. When cocrystal dissolves and the maximum concentration in solution is reached, drug crystallization occurs, crystallization of the stable drug occurs, and then the peak concentration should not be considered the solubility of the cocrystal [41]. Kumineck and collaborators describe the “supersaturation index” (SI) as the advantage of the solubility of the cocrystal in supersaturation in relation to the drug. SA is an indicator of the potential for conversion of cocrystals into the constituent drug (drug precipitation) when the cocrystal comes into contact with solution, such as during dissolution or pharmaceutical processes [41].

The extent to which dissolution takes place under a given set of experimental conditions refers to the solubility of a given solute in a solvent. Solubility is one of the most important characteristics of pharmaceutical cocrystals [42]. The thermodynamic method analyzes the solubilization process of cocrystals using vertex of a triphasic domain concentrations, evaluating the equilibrium of the cocrystal and the drug in solution, and uses its concentration for application in the formulas of thermodynamic solubility and eutectic constant of the cocrystal, which are fundamental to obtain the solubility of cocrystals [42,45]. The solubility of the NFX–INA cocrystal under vertex of a triphasic domain conditions had never been evaluated before. The authors who developed the cocrystal used the technique described by Higuchi and Connoras for solubility evaluation, which provided them with the result that the cocrystal would be approximately 3 times more soluble than NFX [14]. By applying this technique for evaluating the solubility of the cocrystal at the vertex of a triphasic domain, it was possible to verify even more advantageous results. There was an increase of 8.38 times in the solubility in water and 6.41 times in FaSSIF, using a fundamental technique to obtain the solubility of cocrystals. The dissolution under supersaturating conditions showed that the cocrystal dissolves more than the pure drug, which may increase the bioavailability of NFX. Additional studies are necessary to confirm this result.

The *K_eu_* value was used to evaluate the solubility and stability of the cocrystal relative to the pure drug. In water, the *K_eu_* obtained a value of 70.2. In FaSSIF, the value was 41.1. As already mentioned, when it comes to cocrystals of stoichiometric ratio 1:1, the *K_eu_* > 1 values indicate thermodynamic instability of the cocrystal but higher solubility towards the drug, while *K_eu_* < 1 values indicate higher thermodynamic stability and lower solubility of the cocrystal towards the drug. Since the values obtained in water and FaSSIF were greater than 1, this indicates that the cocrystal is more soluble and less stable than the pure drug in both media. The values found are higher than 1 due to the solubility advantage of the cocrystal over the drug, since the higher the *K_eu_* value, the greater its advantage [45]. It is also observed that *Keu* is lower in FaSSIF than in water, which corroborates with the solubility equation result, in which the solubility of the cocrystal in FaSSIF is lower than the solubility of the cocrystal in water.

According to the authors who standardized the microdilution test with the help of TTC, the visual reading becomes reliable, but it requires care in its execution. The MIC of compounds that present similar placement to formazan, resulting from the reduction in TTC, cannot be determined. Also, it is important to point out that the visual reading only allows the determination of the MIC, while the quantification of the microorganisms to determine the IC50% and IC90% is only possible from the spectrophotometric readings. This colorimetric method is relevant since it can be applicable to test the most diverse types of compounds with antimicrobial activity, as well as bringing an innovation since no colorimetric method described in the literature proposes the conversion of absorbance values into CFU/mL [29].

The MICs were determined by visual readings, considering the last concentrations with no development of pink coloration, which resulted from the reduction in TTC by microbial metabolism. Table 3 showed that the cocrystal presented an advantageous antimicrobial activity when compared to the NFX and the physical mixture in the three bacteria analyzed. In *Escherichia coli* and *Staphylococcus aureus* bacteria, the MIC of the cocrystal corresponded to half of the MIC observed in the NFX and physical mixture, while, in the bacterium *Pseudomonas aeruginosa*, the MIC of the cocrystal was eight times lower than the MIC of NFX and physical mixture. The antimicrobial activity of the NFX was also enhanced using the cocrystallization technique with the INA coformer. It is also possible to evaluate that the observed results of the NFX and the physical mixture are equal in each of the three bacteria analyzed, which demonstrates that the antimicrobial action was actually enhanced by the cocrystal formation and not only by the presence of INA in the sample.

The visual reading only allows determination of the MIC, while the quantification of microorganisms is only possible from spectrophotometer readings [29]. Therefore, to determine the IC50% and IC90%, the spectrophotometric reading was performed. Table 4 shows that, in the three bacteria analyzed, the cocrystal presented lower IC50% and IC90% results and, therefore, more advantageous than the results observed for the NFX and the physical mixture of NFX and INA.

Making a relationship between the cocrystal reproduced in this article, which followed the process previously described by Basavoju and collaborators [14], and the other NFX cocrystals described in the literature so far [15,16,17,18,19,20], we can mention that most of them also used organic solvents for its preparation, such as chloroform [17], toluene [18], and ethanol [20], which is, therefore, considered a common practice among authors. When evaluating the DSC/TG results, it can be observed that, after desolvation of the cocrystal at ~110 °C, we have a temperature interval until its melting occurs at 143 °C, which suggests the hypothesis of the existence of an anhydrous cocrystal between these two temperatures that could be isolated and obtained through the use of thermal analysis techniques, offering a possible proposal for an NFX–INA cocrystal without the presence of the CHCl_3_ solvent and an interesting topic for future studies.

The comparison of the solubility of NFX cocrystals (Table 1) is difficult, since different solubility techniques were used and different pHs were studied. We propose the stability of cocrystals, described in Table 1, by analyzing the melting point of cocrystals formed in the DSC results provided by the authors and comparing them with the melting point of the pure drug. DSC is a technique used for characterization, but it is also a means of checking stability [50]. Usually, cocrystals with a melting point lower than the melting point of the drug are less stable, which can be an expected result, since less stable cocrystals tend to be more soluble, and this is what is expected from a pharmaceutical cocrystal [45].

We can also emphasize the lack of antimicrobial evaluation tests of NFX cocrystals mentioned in Table 1. Only the article by Prashar et al. [20] presents such a result and, as in our work, the results of these authors demonstrated that the cocrystal presented a better antimicrobial activity with a lower dose than the drug, which may indicate better pharmacological action [20].

## 5. Conclusions

The NFX–INA–CHCl_3_ cocrystal was formed by using the solvent evaporation technique, and the confirmation of the presence of the new crystalline structure was achieved by performing solid state characterization techniques. The application of the techniques for evaluating dissolution under supersaturation conditions and solubility at the vertex of triphasic domain demonstrated that the cocrystal proved advantageous in water and in FaSSIF when compared to NFX. Furthermore, better antimicrobial action of cocrystal was observed for *Escherichia coli*, *Staphylococcus aureus*, and *Pseudomonas aeruginosa* when compared to NFX and the physical mixture of NFX and INA. These results show that the NFX cocrystal has biopharmaceutical and microbiological advantages when compared to the NFX drug, and, for this reason, it is necessary to continue the in vivo studies of this cocrystal.

## Figures and Tables

**Table 1:** Norfloxacin cocrystals described in the literature.

Coformer	Cocrystallization Technique	Specificities	Improvement in Solubility (mg mL^−1^)	Solubility Assessment Technique	Stability *	Reference
Isonicotinamide	Solvent evaporation	Solvated cocrystal (CHCl_3_)	0.59 ± 0.01(water)	Apparent aqueous solubility	Less stable	Basavoju et al. 2006 [14]
Saccharin	Solvent-assisted mechanochemistry	Cocrystal with an organic counter ion	-	-	-	Velaga et al. 2008 [15]
-	Heteroassociation in the solid state	Heteroassociation with Ciprofloxacin	-	-	More stable	Vitorino et al. 2013 [16]
Riboflavin	Solvent-assisted mechanochemistry	Chloroform as a solvent	-	-	-	Ferreira et al. 2019 [17]
Resorcinol	Solvent-assisted mechanochemistry	Uses Toluene as a solvent	2.64 ± 0.39 (pH 7.5)	Shake-flask method	Less stable	Fael et al. 2022 [18]
Picolinic Acid	Mechanochemistry in mill	-	2.57 ± 0.01 (water); 1.91 ± 0.7 (pH 3); 0.7 ± 0.09 (pH 6.1); 0.6 ± 0.01 (pH 8.5)	Shake-flask method	More stable	Ferreira et al. 2023 [19]
Isonicotinic Acid	Mechanochemistry in mill	-	28.98 ± 0.02 (water); 0.73 ± 0.02 (pH 3); 1.62 ± 0.08 (pH 6.1); 0.59 ± 0.1 (pH 8.5)	Shake-flask method	More stable	Ferreira et al. 2023 [19]
Nicotinamide	Solvent-assisted mechanochemistry	Ethanol as a solvent	28.59 ± 0.2 (pH 1.2); 14.39 ± 0.3 (pH 6.8)	Apparent solubility analysis	Less stable	Prashar et al. 2023 [20]
Cinnamic acid	Solvent-assisted mechanochemistry	Ethanol as a solvent	15.50 ± 0.2 (pH 1.2); 10.05 ± 0.2 (pH 6.8)	Apparent solubility analysis	Less stable	Prashar et al. 2023 [20]
Sorbic acid	Solvent-assisted mechanochemistry	Ethanol as a solvent	13.25 ± 0.2 (pH 1.2); 9.21 ± 0.2 (pH 6.8)	Apparent solubility analysis	Less stable	Prashar et al. 2023 [20]

* In comparison to the pure NFX.

**Table 2:** Results of cocrystal solubility at the vertex of the triphasic domain in water and FaSSIF at 48 and 72 h.

		[NFX]me (mM)	[INA]me (mM)	Cocrystal Solubility (mM)	Solubility Advantage	pH
Water	48 h	1.72 ± 0.09	120.77 ± 3.23	14.41	8.38	7.05
	72 h	1.79 ± 0.20	124.48 ± 2.15	14.91	8.35	7.05
FaSSIF	48 h	4.92 ± 0.02	202.34 ± 7.63	31.56	6.41	6.61
	72 h	4.93 ± 0.14	196.31 ± 5.18	31.10	6.31	6.56

**Table 3:** MICs of NFX, cocrystal, and physical mixture for the microorganisms *Escherichia coli*, *Staphylococcus aureus*, and *Pseudomonas aeruginosa*.

Microorganisms	MIC Norfloxacin (µg mL^−1^)	MIC Cocrystal (µg mL^−1^)	MIC Physical Mixture (µg mL^−1^)
*Escherichia coli* (ATCC 8738)	0.156	0.078	0.156
*Staphylococcus aureus* (ATCC 6538)	2.500	1.250	2.500
*Pseudomonas aeruginosa* (ATCC 9027)	1.250	0.156	1.250

**Table 4:** IC50% and IC90% of NFX, cocrystal, and PM for the microorganisms *Escherichia coli*, *Staphylococcus aureus*, and *Pseudomons aeruginosa*.

Microorganisms	Inhibitory Concentration	Norfloxacin (µg mL^−1^)	Cocrystal (µg mL^−1^)	Physical Mixture (µg mL^−1^)
*Escherichia coli* (ATCC 8738)	CI50%	0.182	0.064	0.082
	CI90%	0.703	0.210	0.345
*Staphylococcus aureus* (ATCC 6538)	CI50%	0.648	0.269	0.746
	CI90%	1.649	0.738	1.934
*Pseudomonas aeruginosa* (ATCC 9027)	CI50%	0.659	0.090	0.318
	CI90%	1.718	0.166	0.720

## Data Availability

Data available under request for the authors.

## Supplementary Materials

The following supporting information can be downloaded at: https://www.mdpi.com/article/10.3390/pharmaceutics15092211/s1, Figure S1: Photomicrographs obtained using a scanning electron microscope (SEM) model Vega 3 (Tescan) with a secondary electron detector (SE) of the Everhart-Thornley type, with a positive potential front grid and a 5 kV voltage filament. Data processing was performed using the VegaTC® software. (a) corresponds to Norfloxacin, (b) to Isonicotinamide and (c) to the cocrystal; Figure S2: X-ray powder diffraction (XRPD) of the solid phase from the eutectic point in water. The diffractograms correspond to NFX, NFX-INA cocrystal and evaluation of the solid phase of the eutectic point after 48 and 72 h. In blue we observe the points corresponding to NFX, while in red we observe the points corresponding to the NFX-INA cocrystal; Figure S3: X-ray powder diffraction (XRPD) of the solid phase from the eutectic point in FaSSIF. The diffractograms correspond to NFX, NFX-INA cocrystal and evaluation of the solid phase of the eutectic point after 48 and 72 h. In blue we observe the points corresponding to NFX, while in red we observe the points corresponding to the NFX-INA cocrystal; Figure S4: Map of the plate used for each bacterium in the microbiological assays. In purple we observe the samples referring to NFX, in green the samples referring to the NFX-INA cocrystal and in orange the samples referring to the physical mixture. From column 10 we observe the NFX blanc (light blue), the cocrystal blanc (pink), the PM blanc (brown), the negative control blanc (red), the positive control (dark green), the negative control (dark blue) and the coformer control (black); Figure S5: *Escherichia coli* bacteria evaluation plate. The development of the reddish color is due to the reduction of TTC by microbial metabolism. Being considered the minimum inhibitory concentration (MIC) the last concentrations, in triplicate, where there was no development of this coloration for the microorganism; Figure S6: *Pseudomonas aeruginosa* bacteria evaluation plate. The development of the reddish color is due to the reduction of TTC by microbial metabolism. Being considered the minimum inhibitory concentration (MIC) the last concentrations, in triplicate, where there was no development of this coloration for the microorganism; Figure S7: *Staphylococcus aureus* bacteria evaluation plate. The development of the reddish color is due to the reduction of TTC by microbial metabolism. Being considered the minimum inhibitory concentration (MIC) the last concentrations, in triplicate, where there was no development of this coloration for the microorganism; Figure S8: *Escherichia coli* growth curves, correlating log CFU/mL with tested antimicrobial concentrations, except for CFU/mL values with negative results. (A) corresponds to the growth curve of Escherichia coli when the antimicrobial used was Norfloxacin, (B) to the cocrystal and (C) to the physical mixture. The growth curve graphs had their determination coefficient (R-squared) and straight line equation determined to obtain the IC50% and IC90% results; Figure S9: *Pseudomonas aeruginosa* growth curves, correlating log CFU/mL with tested antimicrobial concentrations, except for CFU/mL values with negative results. (A) corresponds to the growth curve of Escherichia coli when the antimicrobial used was Norfloxacin, (B) to the cocrystal and (C) to the physical mixture. The growth curve graphs had their determination coefficient (R-squared) and straight line equation determined to obtain the IC50% and IC90% results; Figure S10: *Staphylococcus aureus* growth curves, correlating log CFU/mL with tested antimicrobial concentrations, except for CFU/mL values with negative results. (A) corresponds to the growth curve of Escherichia coli when the antimicrobial used was Norfloxacin, (B) to the cocrystal and (C) to the physical mixture. The growth curve graphs had their determination coefficient (R-squared) and straight line equation determined to obtain the IC50% and IC90% results.
