# Supplementary material for: Cocrystals Enhance Biopharmaceutical and Antimicrobial Properties of Norfloxacin
Source: Pharmaceutics. 2023 Aug 26;15(9):2211. doi: 10.3390/pharmaceutics15092211 (PMC10536922; doi:10.3390/pharmaceutics15092211)
Supplement: Supplementary file 1 [file pharmaceutics-15-02211-s001.zip › pharmaceutics-2523951-supplementary.pdf]

Cocrystals Enhance Biopharmaceutical and Antimicrobial Properties of Norfloxacin

Supplementary Material

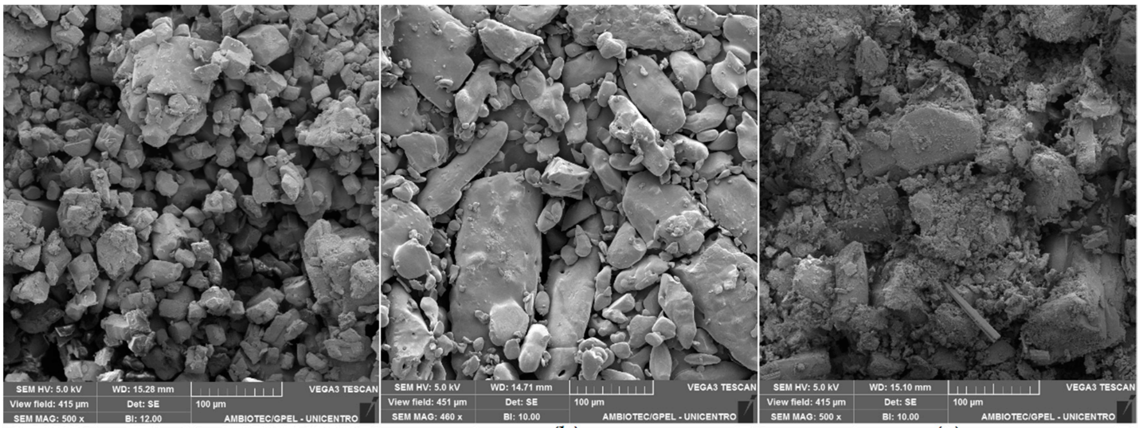

(a) (b) (c)

Supplementary Figure S1. Photomicrographs obtained using a scanning electron microscope (SEM) model Vega 3 (Tescan) with a secondary electron detector (SE) of the Everhart-Thornley type, with a positive potential front grid and a 5 kV voltage filament. Data processing was performed using the VegaTC® software. (a) corresponds to Norfloxacin, (b) to Isonicotinamide and (c) to the cocrystal.

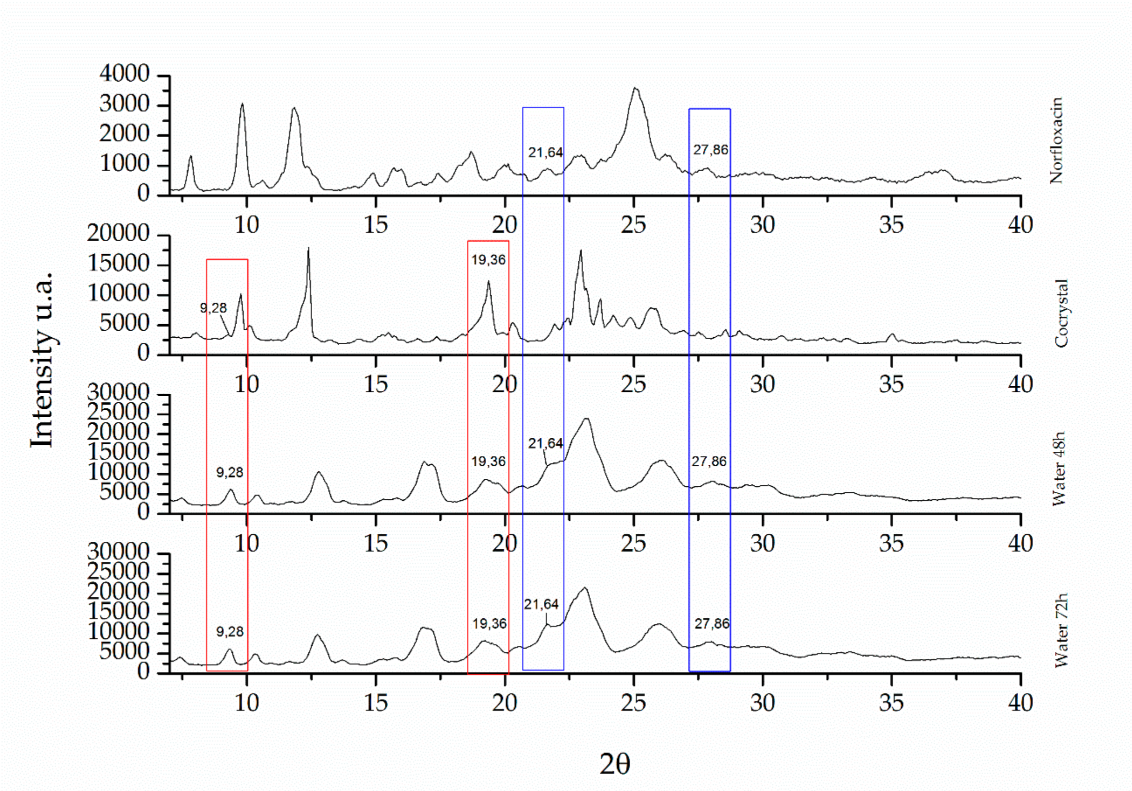

Supplementary Figure S2. X-ray powder diffraction (XRPD) of the solid phase from the eutectic point in water. The diffractograms correspond to NFX, NFX-INA cocrystal and evaluation of the solid phase of the eutectic point after 48 and 72 hours. In blue we observe the points corresponding to NFX, while in red we observe the points corresponding to the NFX-INA cocrystal.

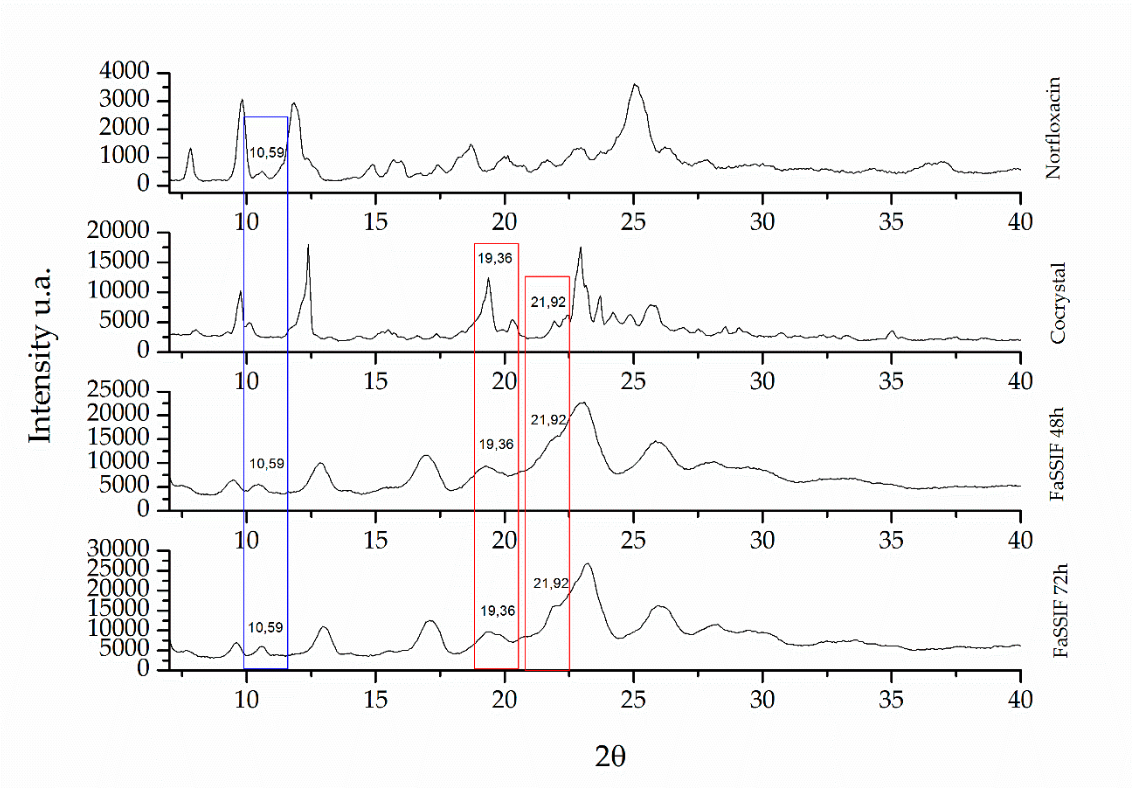

Supplementary Figure S3. X-ray powder diffraction (XRPD) of the solid phase from the eutectic point in FaSSIF. The diffractograms correspond to NFX, NFX-INA cocrystal and evaluation of the solid phase of the eutectic point after 48 and 72 hours. In blue we observe the points corresponding to NFX, while in red we observe the points corresponding to the NFX-INA cocrystal.

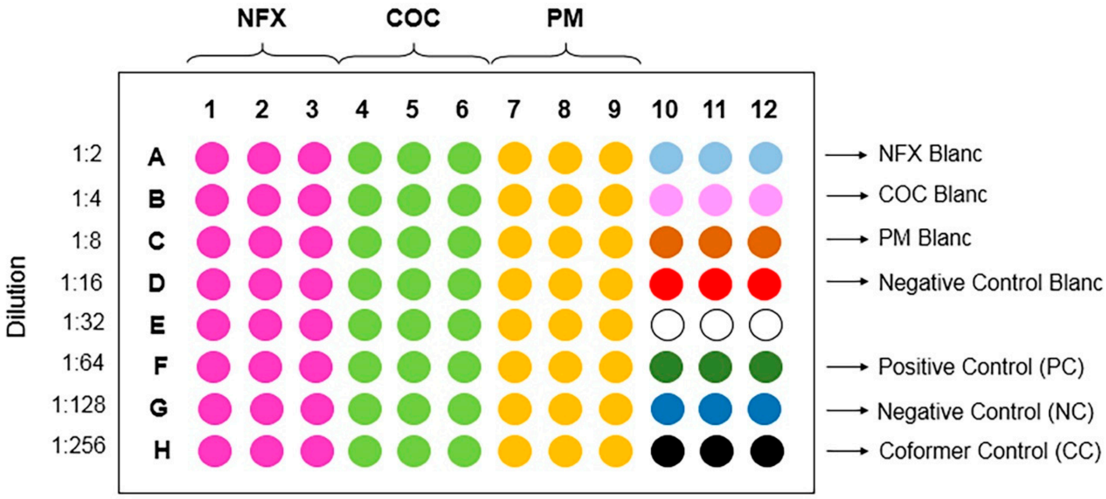

Supplementary Figure S4. Map of the plate used for each bacterium in the microbiological assays. In purple we observe the samples referring to NFX, in green the samples referring to the NFX-INA cocrystal and in orange the samples

referring to the physical mixture. From column 10 we observe the NFX blanc (light blue), the cocrystal blanc (pink), the PM blanc (brown), the negative control blanc (red), the positive control (dark green), the negative control (dark blue) and the coformer control (black).

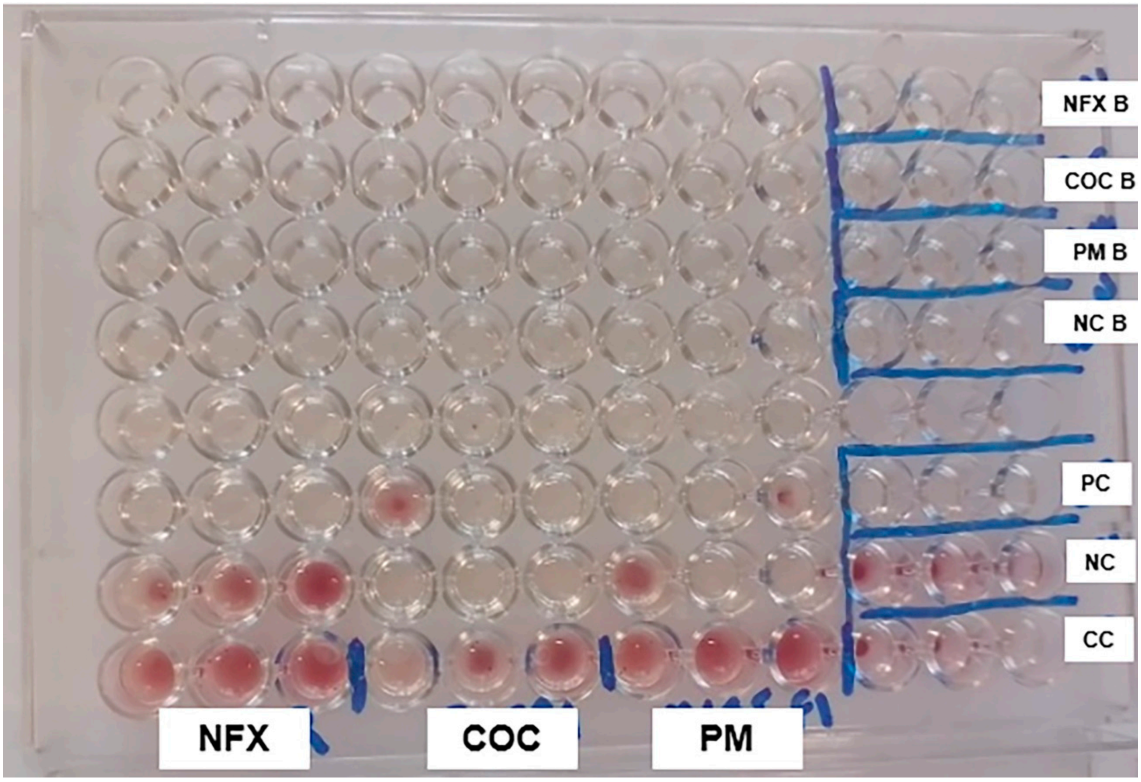

Supplementary Figure S5. *Escherichia coli* bacteria evaluation plate. The development of the reddish color is due to the reduction of TTC by microbial metabolism. Being considered the minimum inhibitory concentration (MIC) the last concentrations, in triplicate, where there was no development of this coloration for the microorganism.

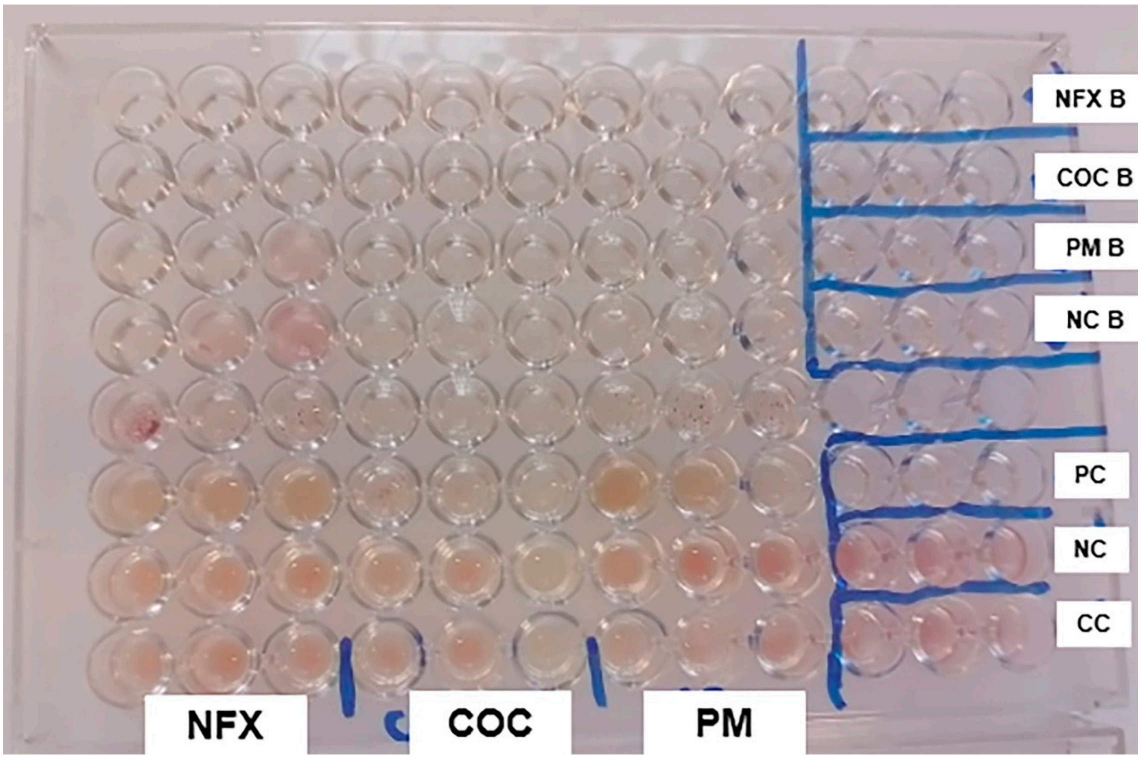

Supplementary Figure S6. *Pseudomonas aeruginosa* bacteria evaluation plate. The development of the reddish color is due to the reduction of TTC by microbial metabolism. Being considered the minimum inhibitory concentration (MIC) the last concentrations, in triplicate, where there was no development of this coloration for the microorganism.

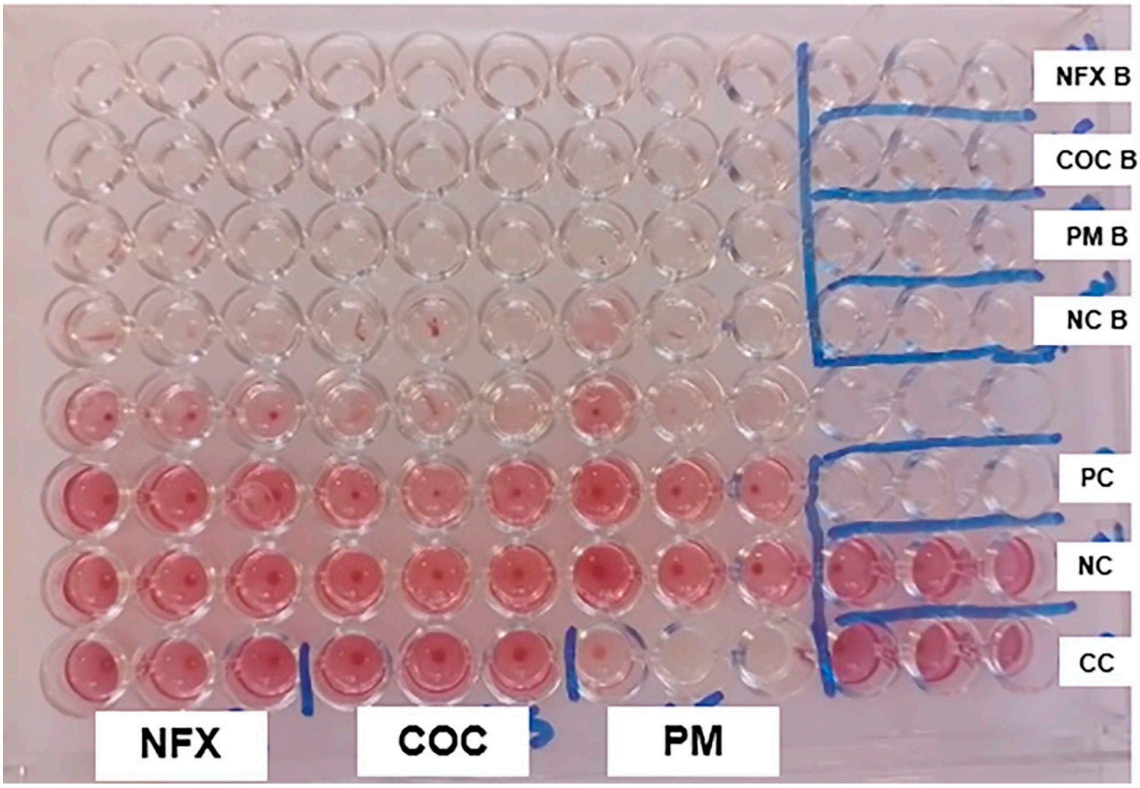

Supplementary Figure S7. *Staphylococcus aureus* bacteria evaluation plate. The development of the reddish color is due to the reduction of TTC by microbial metabolism. Being considered the minimum inhibitory concentration (MIC) the last concentrations, in triplicate, where there was no development of this coloration for the microorganism.

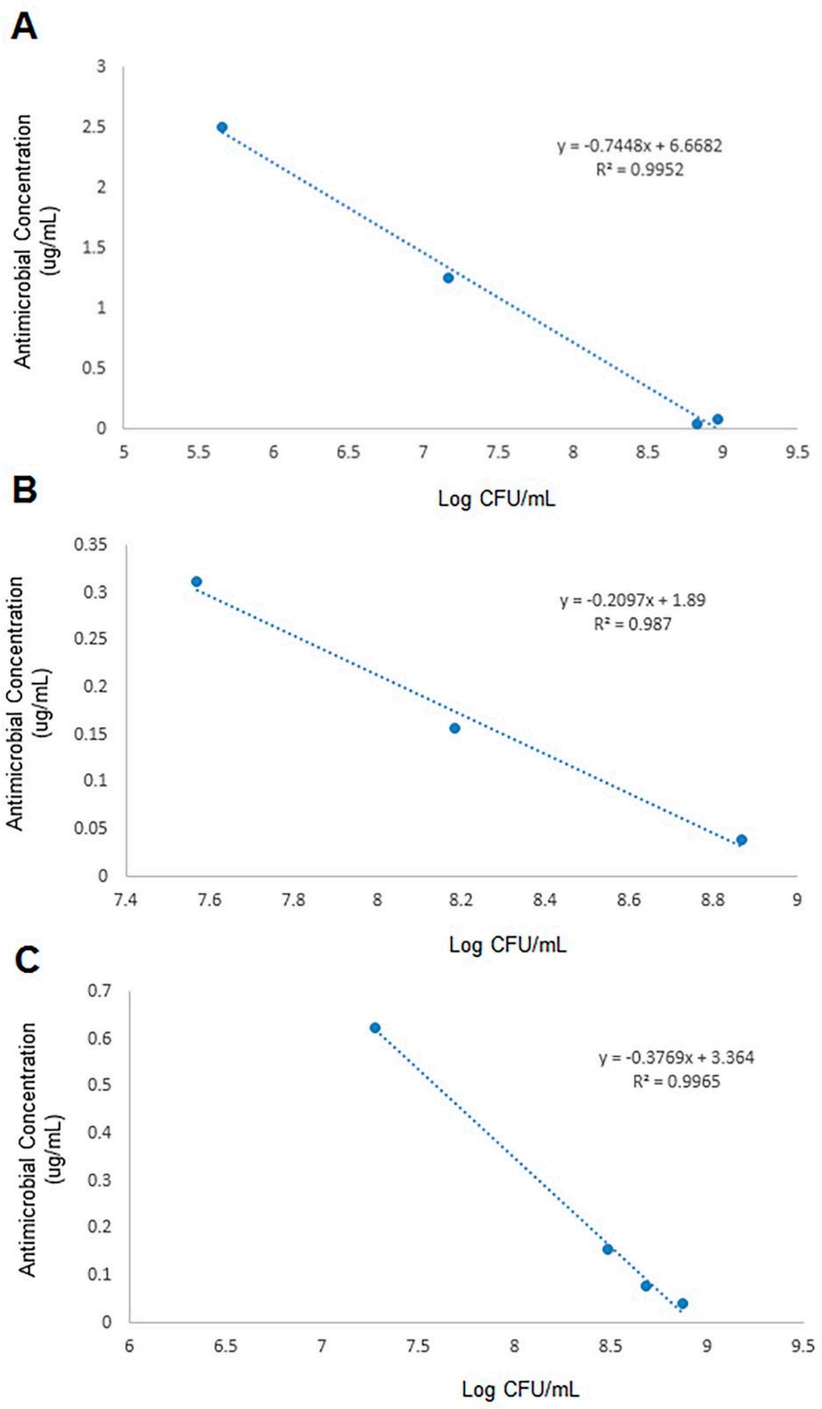

Supplementary Figure S8. *Escherichia coli* growth curves, correlating log CFU/mL with tested antimicrobial concentrations, except for CFU/mL values with negative results. (A) corresponds to the growth curve of Escherichia coli when the antimicrobial used was Norfloxacin, (B) to the cocrystal and (C) to the physical mixture. The growth curve graphs had their determination coefficient (R-squared) and straight line equation determined to obtain the IC50% and IC90% results.

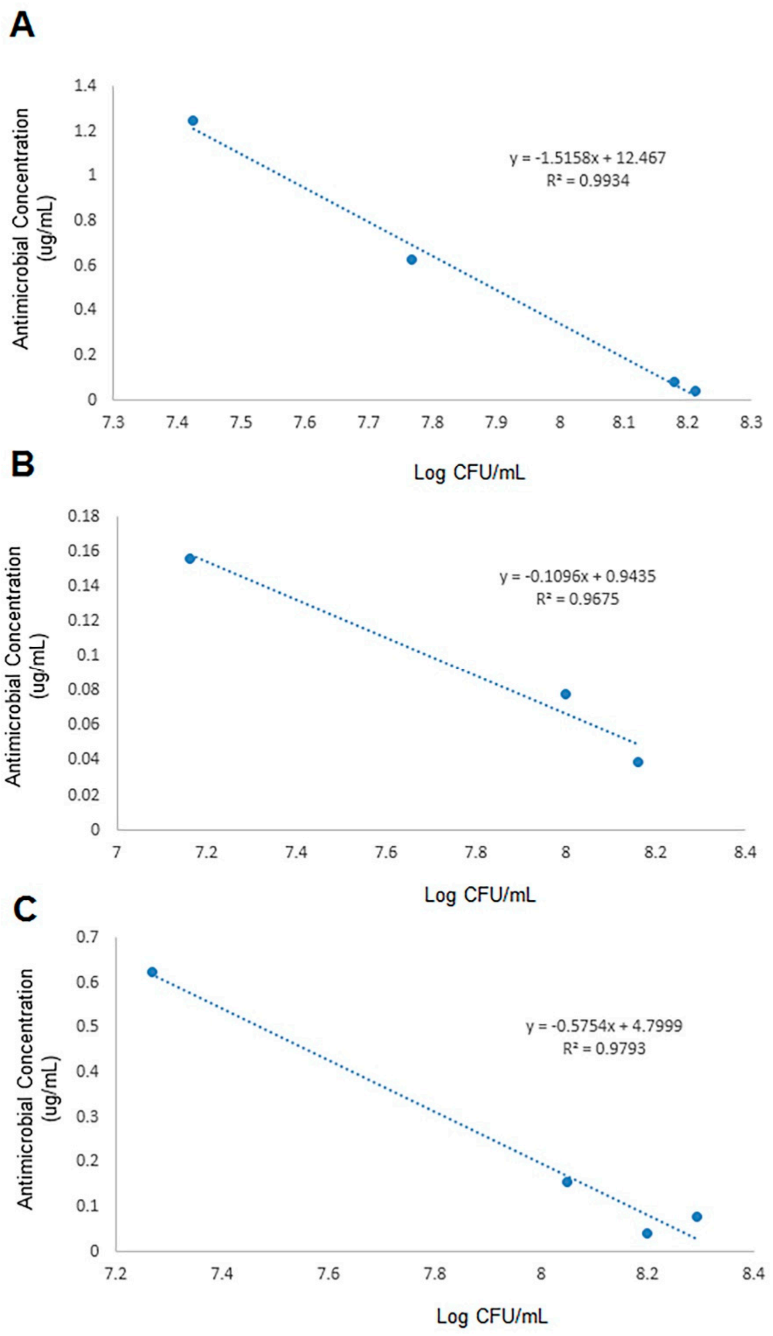

Supplementary Figure S9. *Pseudomonas aeruginosa* growth curves, correlating log CFU/mL with tested antimicrobial concentrations, except for CFU/mL values with negative results. (A) corresponds to the growth curve of Escherichia coli when the antimicrobial used was Norfloxacin, (B) to the cocrystal and (C) to the physical mixture. The growth curve graphs had their determination coefficient (R-squared) and straight line equation determined to obtain the IC50% and IC90% results.

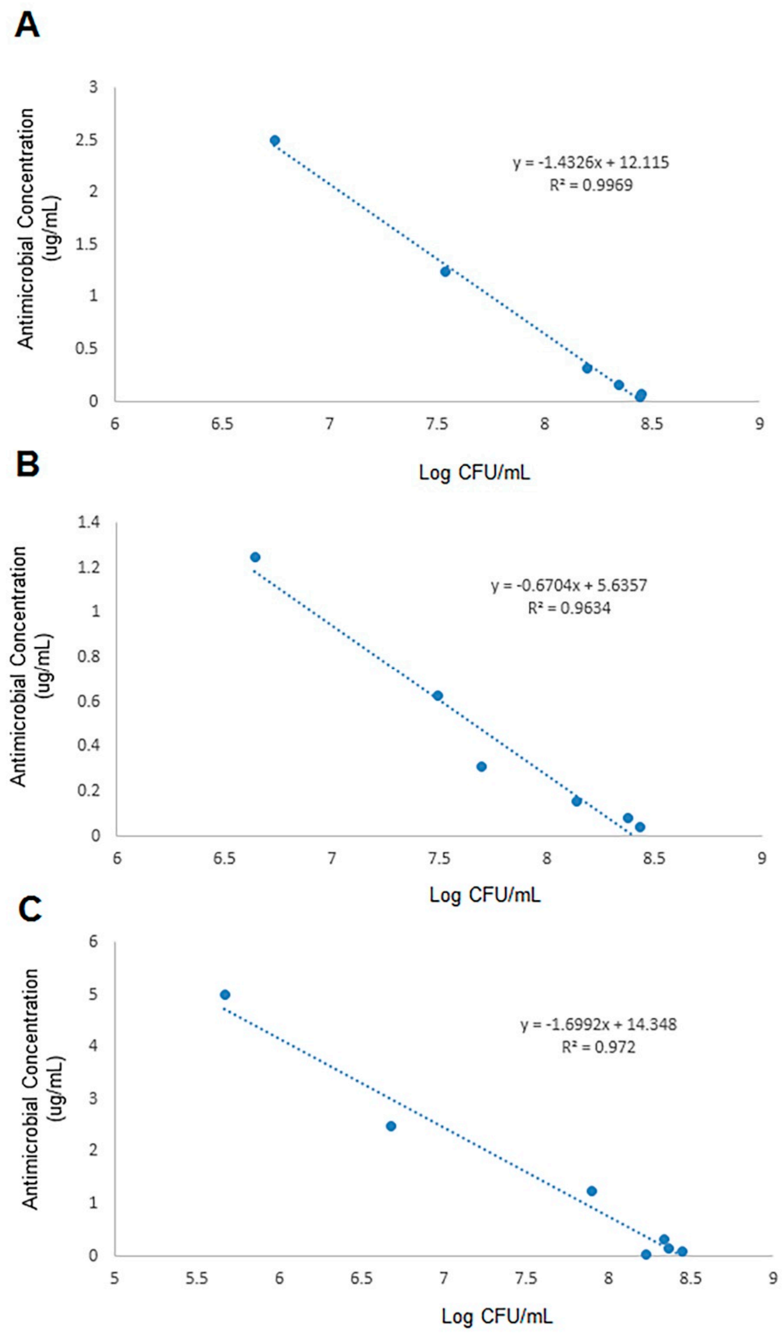

Supplementary Figure S10. *Staphylococcus aureus* growth curves, correlating log CFU/mL with tested antimicrobial concentrations, except for CFU/mL values with negative results. (A) corresponds to the growth curve of Escherichia coli when the antimicrobial used was Norfloxacin, (B) to the cocrystal and (C) to the physical mixture. The growth curve graphs had their determination coefficient (R-squared) and straight line equation determined to obtain the IC50% and IC90% results.
